# The Changes in the Morphology of Bi–Sb System under Centrifugal Force at Room Temperature

**DOI:** 10.3390/ma11071065

**Published:** 2018-06-23

**Authors:** Bartek Wierzba, Wojciech J. Nowak

**Affiliations:** Department of Materials Science, Faculty of Mechanical Engineering and Aeronautics, Rzeszow University of Technology, Powstancow Warszawy 12, 35-959 Rzeszow, Poland; w.nowak@prz.edu.pl

**Keywords:** gravity, sedimentation, high speed rotation, voids formation, room temperature

## Abstract

In this paper, the morphology evolution in a Bi–Sb system under the centrifugal force at room temperature is discussed. The rotation speed was 35,000 rotations per minute and the length of the arm (radii) was 88 mm. It is shown, that after the centrifugation the phases become oriented in the direction parallel to the direction of the centrifugal force. Moreover, the voids move in the direction opposite to the centrifugal force direction. The movements of such voids result in their coalescence and finally lead to formation of cracks at the interface between ordered and disordered part of the sample.

## 1. Introduction

Except the chemical potential, temperature gradient, and electric field also strong gravitational field causes the migration of the atoms due to the differences in the atomic mass of the alloying elements (so called “sedimentation”). The pressure and temperature are thermodynamic variables that affects atoms statistically. Gravity can be characterized as a field-state variable, like magnetic and electric fields. This variable acts directly on atoms in materials. The sedimentation process of Au atoms in elemental metals K, Pb, and In under maximum acceleration fields of 1–2 × 10^5^ G with low melting temperatures was investigated by Barr and Smith and Anthony in the 1970s [1,2]. One of the first ultracentrifuge apparatus was developed by Mashimo et al in 1996. It can for a long time generate an acceleration field up to 10^6^ × G at high temperatures [3]. It was shown, that the atomic scale is disturbed by a strong gravitational field, which can induce sedimentation of atoms or structural change [4,5]. The centrifugal forces can form an atomic-scale graded structure in condensed matter [6] influencing the isotopes segregation [7]. The sedimentation of the substitutional solute atoms of different elements [8,9,10] was studied by Mashimo e.g., ultracentrifuge experiments on elemental selenium to examine the sedimentation of isotope atoms in liquid matter at high temperature [11]. The Bi_70_Sb_30_ (at. %) alloy was ultracentrifuged under an ultra-strong gravitational field of (0.79–0.96) × 10^6^ G at a temperature of 220–240 °C for 85 h [6]. The components concentration was changed under the high temperature. Moreover, the deformation twins with disorientation of about 90° were observed in the low-gravitational region where grain refinement had not occurred [12].

The materials science studies using microgravity has been performed to fabricate high quality materials or single crystal protein, etc. by preserving convection and mixing uniformly. On the other hand, a strong gravity enables control of compositions or structure, crystalline state, etc., because small differences in atomic weight influence the concentrations, lattice ordering or crystalline state under such strong gravitational field. By the sedimentation, we can aim at materials control in atomic scale, and realize discovery of new materials or new properties. It is expected that the strong gravitational field will be used as a new method of atomic-scale materials processing to control the composition, impurities, nanostructure, and interface structure of materials, and to concentrate isotopes [13]. Therefore, in the present paper, the influence of the centrifugal force on the Bi–Sb alloy was studied at room temperature to elucidate centrifugation as a potential method for modification of the materials properties.

## 2. Experimental

In the present work a binary model alloy with nominal composition 45% Bi–55% Sb (in wt. %) has been produced. From such cast alloy a rods with 4 mm length and 2 mm diameter were machined. Such prepared samples were tested using ultracentrifugal Schenck (Schenck, Darmstadt, Germany) apparatus at room temperature for 1 h with the rotation speed of 35,000 RPM. The length of the arm was 88 mm. The specimens were fixed in the apparatus arm (rotating disc) using holder made of Ti. The temperature was controlled “in situ” using a thermocouple placed near sample in the holder, namely, thermocouple was positioned 1 mm above rotating disc. Moreover, the temperature was additionally monitored by pyrometer at position of sample mounting. Both measurements show temperature increase up to maximum 1 °C during centrifugation. The samples in as-cast condition as well as after the test were mounted in the thermosetting phenolic hot mounting resin containing carbon filler PolyFast made by Struers. The polymerization was done at temperature 180 °C for 2.5 min. Considering very short time of heating its effect on the microstructure has been neglected in the present work. Metallographic cross sections were prepared by series of grinding and polishing steps, finishing with a fine polishing in SiO_2_ suspension with 0.25 µm granulation. The cross-sections were analyzed in term of microstructure and chemical composition using scanning electron microscope Hitachi S3400N (SEM) (Hitachi, Tokyo, Japan). Phase analyses of the material prior and after ultracentrifugation were performed using an X-ray diffractometer Miniflex II made by Rigaku (Rigaku, Tokyo, Japan). As a X-ray source filtered copper lamp (CuK_α_, λ = 0.1542 nm), with a voltage of 40 kV was used. The 2θ angle range varied between 20°–100° and step size was 0.02°/3 s. Phase composition was determined using the Powder Diffraction File (PDF) developed and issued by the ICDD (The International Center for Diffraction Data).

## 3. Results

**Figure 1:**
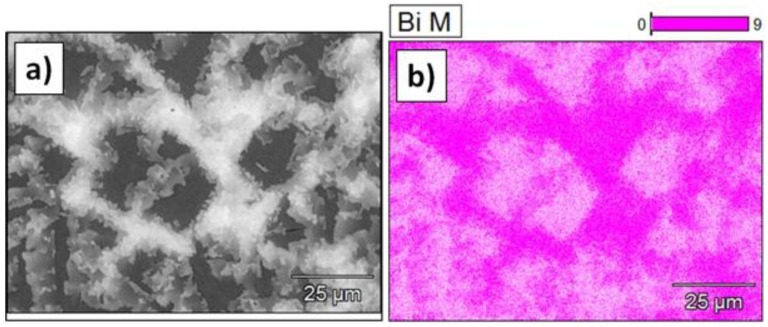

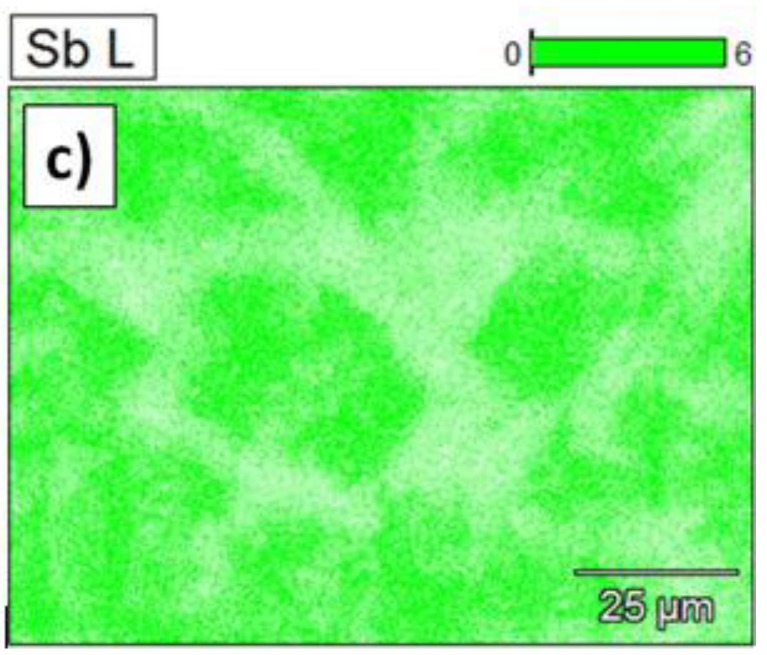
The SEM/BSE (scanning electron microscope equipped with backscattered electrons detector) image (**a**); SEM/EDX (scanning electron microscope/Energy Dispersive X-Ray Spectroscopy) elemental concentration maps of Bi (**b**); and Sb (**c**) performed on the Bi–Sb alloy in the as-cast condition. More intensive color indicates higher element concentration.

The SEM/EDX (scanning electron microscope/Energy Dispersive X-Ray Spectroscopy) measurement of the alloy chemical composition showed good agreement with the nominal one, namely the measured content of Bi was 54.5 wt. % and Sb 45.5 wt. % which represent 67.3 and 32.7 at. %, respectively. The microstructure of the studied alloy in the as-cast condition is shown in Figure 1. It is clear that the alloy contains two phases.

The SEM elemental maps unambiguously showed that the light phases are enriched in Bi while the darker one contains more Sb (Figure 1). Moreover, the results of SEM/EDX point analysis revealed that the lighter phases shown in Figure 1a consist of 64 at. % of Bi and 36 at. % of Sb, while the chemical composition of the dark phase is 15 at. % of Bi and 85 at. % of Sb. The two phases formation the most likely occurred during the rapid cooling process during casting.

**Figure 2:**
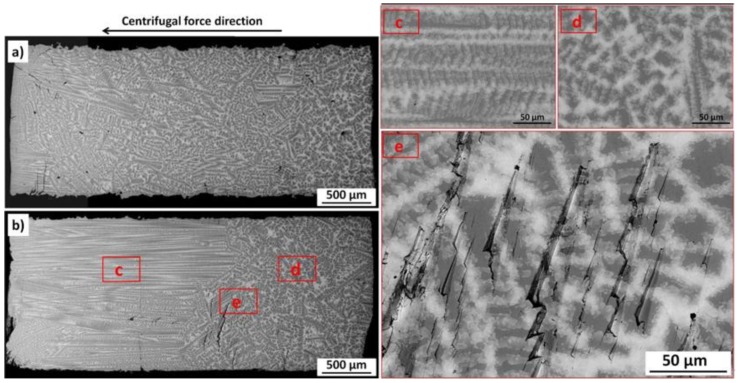
SEM/BSE images of the cross-sections of Bi–Sb alloy showing microstructure in: (**a**) as-cast condition; (**b**) after centrifugation; (**c**) high magnification of ordered part; (**d**) high magnification of disordered part; and (**e**) high magnification of crack at disordered and ordered parts interface.

Figure 2 shows the comparison of the alloy microstructure in the as-cast condition (Figure 2a) and after the experiment (Figure 2b).

The microstructure of the alloy in the as-cast condition showed random distribution and/or orientation of the phases over the whole investigated area. The microstructure of the alloy after experiment showed the orientation parallel to the centrifugal force direction in the outer part of the sample (left side of Figure 2b). The SEM/BSE (scanning electron microscope equipped with backscattered electrons detector) images performed using higher magnification of the ordered and disordered parts of the samples (Figure 2c) showed, that mainly “light” phases (containing roughly 64 at. % of Bi) formed some kind of lines. 

Moreover, the specimen shows inner cracks after centrifugation (Figure 2e). This fact can be correlated with the movement and coalescence of voids in the opposite direction to the direction of centrifugal force. Accumulation of voids causes formation of cracks (Figure 2e).

**Figure 3:**
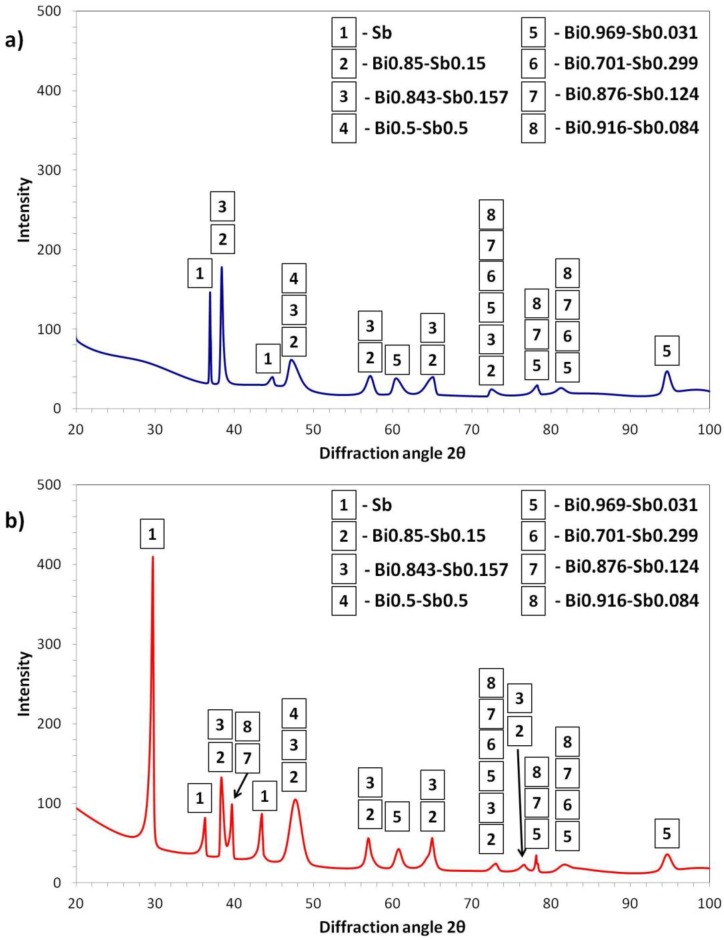
XRD (X-ray diffraction) patterns obtained on the cross-sections of Bi–Sb alloy in: (**a**) as-cast condition and (**b**) after centrifugation.

To investigate the phase composition of studied alloy before and after centrifugation an XRD (X-ray diffraction) analysis has been performed. The obtained XRD patterns are shown in Figure 3. It is clear that the XRD patterns obtained from the sample in as-cast condition (Figure 3a) and after centrifugation (Figure 3b) substantially differs. For the material in the as-cast condition, a number of phases, mainly consisting of around 85% of Bi and 15% of Sb are visible. Moreover, peaks of Sb are present at around 37° and 45°. After centrifugation, an additional, very strong signal coming from pure Sb occurred at about 29°. Except for an additional signal from Sb, other peaks at 39° and 76° occurred after the test. These peaks were identified to come from the phases containing mainly Bi (of about 85%) and small amounts of Sb (roughly 15%). Moreover, after centrifugation, the intensities of the peaks are higher. This two observations leads to the conclusion that during centrifugation part of the phases containing both Bi and Sb becomes separated leading to the increase in the amount of pure Sb as well as phases containing more Bi in the chemical composition (compare Figure 3a,b).

Under the strong centrifugal force heavier atoms (Bi in the present case) are more affected by the centrifugal force and might migrate along the centrifugal force direction. This in turn, is most probably responsible for the observed changes in the crystallographic structures and sample morphology. However, it has to be highlighted that at room temperature, this movement is not as sufficient as it is at elevated temperatures and does not lead to the gradient in Bi and Sb concentration, as observed by Huang et al. [6] at 220–240 °C. In the case of the present study, the centrifugation leads to the microstructural changes which most probably are caused by the stronger influence of centrifugal force on Bi atoms. Moreover, this change is present only in two thirds of the sample length (Figure 2b). The ordered part of the sample was places in the very outer part of the rotating arm, where the gravitational force was the highest one. Apparently, at one third of the sample length, the centrifugal force was not enough high to overcome the large energy of chemical potential (due to the smaller radius). As the result, partial reorganization of the sample microstructure is observed.

## 4. Discussion and Conclusions

It is well known, that under the pressure, the lattice shrinks isotropically. This situation is described by the equation of the state of matter. In a strong gravitational field, the heavier atoms are subjected to a stronger force along the gravitational direction. The lighter atoms are forced in the opposite direction. The reason is that there are different body forces acting on the respective atoms owing to their different atomic weights. This can cause the structural changes, which can be different than those caused by high pressure and/or high temperature.

In the present study the following observation have been made:

- The binary Bi–Sb alloy showed the formation of two phases: one enriched in Bi and the second enriched in Sb. Formation of the two phases most likely originate due to the rapid cooling during alloy casting;
- Under centrifugal force the phases become oriented in the direction parallel to the direction of the centrifugal force;
- Ordering of the phases results in void movement in the direction opposite to the centrifugal force direction. The movements of such voids result in their accumulation and formation of cracks at the interface between ordered and disordered parts of the sample;
- Changes in phase composition after centrifugation was observed as well. Namely, more peaks of Sb phase are observed as well as higher intensities of the peaks from Bi enriched phases, e.g., Bi0.85–Sb0.15.

The results obtained in the present study clearly demonstrate that the centrifugal force influences not only the morphology but also the structure and texture of the Bi–Sb system even at room temperature. Considering the fact that the centrifugation was performed at temperatures higher than 0.4 Tm, one can expect that the diffusion should occur during the process. Thus the diffusion potential is influenced by the chemical and mechanical potentials. The mechanical potential is defined by the pressure generated during the process:

(1) $$\mu_{i}^{m}=-V_{i}\cdot r\cdot \omega^{2}\cdot \rho$$

where $\mu_{i}^{m}$—is the mechanical potential, $V_{i}$—volume of the *i*-th component, r—radii, ω—centrifugal velocity, and ρ—density.

In the present work, the SEM/EDS measurements of average chemical composition of ordered and disordered part of the sample after centrifugation do not shows the changes during the process at room temperature. This observation strongly suggests that the main factor causing the changes in the studied system can be short distance diffusion.

In contrast, the phase composition analysis done by XRD clearly shows that under the strong gravitational field at room temperature, the phase composition presented in the as-cast material becomes reconstructed. Moreover, the qualitative results of void formation and crack generation was obtained by Ogata et al. [14]. This means that the centrifugal force influences the chemical potential of the system and changes the diffusion mechanisms.
